# Role of pre-operative anxiety on intraoperative anesthetic requirement and post-operative recovery: An observational study

**DOI:** 10.6026/973206300223449

**Published:** 2026-06-30

**Authors:** Sushil Chand Verma, Ajita Tiwari, Irfan Ahmad Siddiqui

**Affiliations:** 1 Department of Anaesthesia, Birsa Munda Government Medical College, Shahdol, Madhya Pradesh, India; 2 Department of Anaesthesia, Government Medical College, Satna, Madhya Pradesh, India

**Keywords:** Pre-operative anxiety, anesthesia, post-operative recovery, analgesic requirement, APAIS scale

## Abstract

Pre-operative anxiety is commonly observed in patients undergoing surgical procedures and may influence perioperative outcomes. Therefore, it is of interest to evaluate the effect of pre-operative anxiety on intraoperative anesthetic requirements and post-operative recovery outcomes. A prospective observational study was conducted on 100 patients undergoing elective surgeries under general anesthesia, where pre-operative anxiety was assessed using the APAIS scale and correlated with anesthetic consumption and recovery parameters. Patients with higher anxiety levels required significantly greater doses of anesthetic agents, had higher post-operative pain scores and showed delayed recovery with increased analgesic requirements. Thus, data shows that pre-operative anxiety has a measurable impact on anesthetic management and recovery, highlighting the importance of early identification and appropriate management of pre-operative anxiety.

## Background:

Pre-operative anxiety is frequently observed among patients scheduled for surgery, with its prevalence varying widely across different clinical settings [1]. This anxiety usually stems from concerns related to anesthesia, surgical outcomes, post-operative pain and uncertainty regarding recovery [2]. Such psychological stress triggers activation of the hypothalamic-pituitary-adrenal axis and sympathetic nervous system, leading to increased release of stress hormones like cortisol and catecholamines, which may affect perioperative physiological responses [3]. Emerging evidence indicates that heightened levels of anxiety before surgery can influence anesthetic drug requirements by modifying both pharmacokinetic and pharmacodynamic responses [4]. Patients experiencing greater anxiety often require higher doses of anesthetic agents to achieve adequate depth of anesthesia [5]. In addition, anxiety has been associated with increased pain perception, delayed recovery and a higher incidence of post-operative complications, including nausea and prolonged hospitalization [6]. Although previous studies have explored this association, there is still a need for well-structured observational studies to clearly establish the relationship between pre-operative anxiety, intraoperative anesthetic consumption and post-operative recovery outcomes. Therefore it is interest to evaluate these parameters in patients undergoing elective surgical procedures.

## Methodology:

This prospective observational study was conducted in a tertiary care center on 100 patients aged 18-65 years undergoing elective surgeries under general anesthesia. The sample size was determined based on previous similar studies and feasibility during the study period. Pre-operative anxiety was assessed using the APAIS scale one day prior to surgery and patients were categorized into low and high anxiety groups. Standard anesthesia protocols were followed and intraoperative as well as post-operative parameters were recorded for analysis.

## Inclusion criteria:

[1] Patients aged 18-65 years

[2] ASA physical status I and II

[3] Elective surgeries under general anesthesia

[4] Willing to participate and provide consent

## Exclusion criteria:

[1] Psychiatric illness

[2] Chronic use of sedatives or opioids

[3] Emergency surgeries

[4] ASA grade III and above

[5] Refusal to participate

## Statistical analysis:

Data were analyzed using SPSS version 25. Continuous variables were expressed as mean ± standard deviation and compared using the independent t-test. Categorical variables were analyzed using the Chi-square test. Correlation analysis was performed to assess the relationship between anxiety scores and study parameters. A p-value <0.05 was considered statistically significant.

## Ethical approval:

All procedures performed in this study involving human participants were in accordance with the ethical standards with the principles of the Declaration of Helsinki. Written informed consent was obtained from all participants prior to their inclusion in the study.

## Results:

A total of 100 patients were included and divided into high anxiety (n=50) and low anxiety (n=50) groups. Baseline demographic characteristics were comparable between the groups, with no statistically significant differences observed. The demographic profile of patients in both groups was comparable, with no statistically significant differences observed. This indicates that baseline characteristics were well matched, minimizing potential confounding effects on study outcomes (Table 1). A highly significant difference in APAIS scores confirms appropriate classification of patients into high and low anxiety groups, validating the grouping methodology used in this study (Table 2). Patients with higher pre-operative anxiety required significantly greater doses of induction agents, inhalational anesthetics and opioids. This suggests a direct relationship between anxiety levels and increased anesthetic demand during surgery (Table 3). Higher anxiety levels were associated with delayed recovery, increased pain perception and greater post-operative analgesic requirement, indicating a negative impact of anxiety on recovery outcomes (Table 4). Correlation between pre-operative anxiety (APAIS score) and total anesthetic requirement showing a strong positive correlation (Figure 1 - see PDF). Correlation between pre-operative anxiety (APAIS score) and duration of PACU stay showing a strong positive correlation (Figure 2 - see PDF).

## Discussion: 

The present study demonstrates that pre-operative anxiety has a significant impact on intraoperative anesthetic requirements, which is consistent with findings reported in previous studies [7]. The physiological stress response associated with anxiety leads to increased catecholamine release, which may alter anesthetic sensitivity and contribute to higher drug requirements and has also been linked to adverse perioperative outcomes [8]. In our study, patients with higher anxiety levels required greater amounts of induction and maintenance anesthetic agents, as well as opioids. Similar trends have been observed in previous studies [9], suggesting that anxiety contributes to increased anesthetic demand and variability during surgery. This may be explained by enhanced sympathetic activity and altered central nervous system processing in anxious individuals. Post-operative recovery was also adversely affected, with anxious patients showing prolonged recovery times and higher pain scores. These findings are consistent with previous reports indicating that anxiety can intensify pain perception through both psychological and neurophysiological mechanisms [10]. Furthermore, the increased need for post- operative analgesics observed in this study aligns with existing evidence on the relationship between anxiety and pain modulation [5, 6]. In addition, higher levels of pre-operative anxiety have been associated with less favorable perioperative outcomes, including delayed recovery and reduced patient satisfaction [11, 12]. Therefore, early identification and appropriate management of pre-operative anxiety may play a crucial role in improving perioperative outcomes and optimizing anesthetic utilization.

## Conclusion: 

Pre-operative anxiety plays a significant role in influencing intraoperative anesthetic requirements and post-operative recovery outcomes. Patients with higher anxiety levels require increased anesthetic and analgesic doses and tend to have delayed recovery with higher pain perception. Thus, routine assessment and appropriate management of pre-operative anxiety should be considered an essential component of perioperative care to improve patient outcomes and optimize anesthetic utilization.

## Figures and Tables

**Table 1:** Demographic characteristics of study population

**Variable**	**High Anxiety (n=50)**	**Low Anxiety (n=50)**	**p-value**
Age (years)	41.2 ±10.4	39.8 ±9.7	0.48
Gender (M/F)	24/26	27/23	0.56
BMI (kg/m^2^)	25.6 ±3.2	24.9 ±2.8	0.41
ASA I/II	28/22	30/20	0.67

**Table 2:** Pre-operative anxiety scores

**Parameter**	**High Anxiety**	**Low Anxiety**	**p-value**
APAIS Score	18.6 ±2.5	9.8 ±1.7	<0.001

**Table 3:** Intraoperative anesthetic requirement

**Parameter**	**High Anxiety**	**Low Anxiety**	**p-value**
Propofol (mg)	155.2 ±20.5	132.4 ±16.3	<0.01
Isoflurane (%)	1.5 ±0.2	1.2 ±0.2	<0.01
Fentanyl (μg)	115.3 ±12.1	96.5 ±11.4	<0.01

**Table 4:** Post-operative recovery parameters

**Parameter**	**High Anxiety**	**Low Anxiety**	**p-value**
PACU Stay (min)	68.4 ± 14.2	52.1 ± 10.5	<0.01
VAS Score	6.5 ± 1.3	4.7 ± 1.1	<0.01
Analgesic (mg)	85.2 ± 14.6	62.3 ± 12.5	<0.01
